# Supplementary material for: The Mulberry SPL Gene Family and the Response of MnSPL7 to Silkworm Herbivory through Activating the Transcription of MnTT2L2 in the Catechin Biosynthesis Pathway
Source: Int J Mol Sci. 2022 Jan 20;23(3):1141. doi: 10.3390/ijms23031141 (PMC8835075; doi:10.3390/ijms23031141)
Supplement: Supplementary file 1 [file ijms-23-01141-s001.zip › ijms-1524708-supplementary.pdf]

Table S1. The expression profile of *MnSPL* genes in different mulberry tissues.

Gene Name	Root_RPKM	Branch bark_RPKM	Winter bud_RPKM	Male flower_RPKM	Leaf_RPKM
MnSPL1	137.788	85.825	52.193	98.672	82.759
MnSPL2	19.383	35.94	56.325	76.481	45.482
MnSPL3	0	0.316	119.484	9.8	3.921
MnSPL4	31.195	31.452	65.528	146.131	322.719
MnSPL5	1.042	1.525	18.076	20.773	39.895
MnSPL6	1.651	7.633	51.073	47.932	25.367
MnSPL7	39.171	44.249	64.463	67.043	64.948
MnSPL8	1.424	4.619	127.308	81.57	37.424
MnSPL10	0	0.194	21.392	3.057	1.119
MnSPL12	9.43	61.742	36.267	47.49	12.924
MnSPL13	38.844	18.672	27.213	18.193	10.877
MnSPL14	149.26	97.052	52.182	74.611	63.814
MnSPL15	5.915	5.058	100.742	12.171	21.112
MnSPL16A	20.268	43.843	121.371	45.61	47.039
MnSPL16B	2.996	3.207	37.564	61.584	17.729

RPKM: Reads Per Kilobase per Million mapped reads.

Table S2. Primers of *MnSPL* and miR156 used in RT-qPCR and Dual-Luciferase Reporter assay.

Gene Name	Forward Primer (5’-3’)	Reverse Primer (5’-3)
	Primers used in RT-qPCR	
SPL1	ACCTGAAGATTATGCTCGTT	GCTGCTTACTGGCTCATT
SPL2	GTTCATTTATTCTGCTGGTT	TCTTAGGATTCTCGGTTG
SPL4	TAGCGTTGACTTGACCGACT	CATTGACGAGGACGACTGTG
SPL6	GAGCTCTAATCCAATGCGAAAAC	GAATTCGGAGAAATCAAGGTTCC
SPL7	AGGTAAACGGAGTTGTCG	GAACCATCTGGCTGCTAA
SPL8	AGCAGCCAAACCAACAAG	TTGCCTGAAACAATCTAACG
SPL10	GAACGCCAAGGATTACCA	ACATGAAACCGGCTACACT
SPL12	CACCAATGCCAAGGACTA	ATTATGACCAGCCAAACG
SPL13	ACACTCGGATTGTGCTCT	CTGAACTCATGGGCTCTAA
SPL14	TATGGAGGATTCGGATTG	CTACTAGATGCCAGTTTCG
SPL15	ATGCATTTCAGTAGTTGCAGACTCA	CCATATCACTCTTTACGCCCCA
SPL16A	ACTGTGAAAGGCATGTGG	ATTGAAGGGTGTTGGAGG
SPL16B	ATGCAGGGACTATCACAGA	GTCAAACTCCACCAAGGA
CHS2	ATCATGGCAATCGGGACG	TGCTCACTGTTAGTAATACGGAAA
CHI	GGGAAGTCGGTGGAGGAA	CGTCTGCGTCAGTGTAAATC
F3’H	CGCCGTGCTGAGATTTG	CTAACCGCTTCACCCTGTA
FNS	CCCTCACCATCCTGCTC	GCTTTCGGCGGGCTTAT
FLS1	TTCCCGAGATACCCACCGTC	TTGGCGTAAAACTCTTTCTCCT
DFR	TTGTCGGACCGTAAAGATG	GCAGTGGGACCAAGAAAT
LAR	TTTGGGCACGACGTAGAC	GCCATCACCGTAGATTTTG
TT2L1	GAATTACTGGAACACCAATTTAGG	TCTTATTGGACGGTGATGCC

GL3	ACGGGATCATTTCCTTAATTATTCA	GTGAGAAAATAACAAGCTTCAACTGT
TTG1	GGCTCCGCAGAGTTACAGAC	AAAGGCGATGGCGATCCAAT
RPL15	GGCTATGTGATTTACCGTGTT	TTGGTCCAGTATGAGTTGAGA
MnSPL16B	ATGCAGGGACTATCACAGA	GTCAAACTCCACCAAGGA
mno-miR156	gcgcTTGACAGAAGATAGAGAGCAC	Supplied by the kit
mno-miR166b	TCGGACCAGGCTTCATTCCC	Supplied by the kit
MnU6	GGAACGATACAGAGAAGATTAGCATGGC	Supplied by the kit
	Primers used in Dual-Luciferase Reporter assay	
MnSPL2	ATGGAGTGGAATTCAAAAGCTTC	CTACTTGAAGTGTGAGGAATAAAAAG
MnSPL7	ATGGAAGGAGCATCGTCTC	TTGGTGCCGAAGTCTTCTAG
MnSPL14	ATGGAAGAGGTGGGCGC	TTGTGGTGATTGCTTCTGC
MnSPL16A	ATGGAGTGGGACTTGAAGG	CTACTCCAACGAAATCGGAAG
Pro-MnTT2L2	GACGAGAGAAAGAAGGACAG	ATCTTGTGTACTTCAAGGTC

Table S3. The expression profile of *MnSPL* genes in mulberry leaves after herbivory treatment.

Name	*Morus notabili*			*Morus atropurpurea* cv. Guiyou 62		
	CK (RPKM)	B 1 h (RPKM)	Fold change	CK (RE)	B 1 h (RE)	Fold change
SPL1	139.41479	169.66340	1.22	0.27339	0.07557	0.28
SPL2	21.71637	13.46324	0.62	0.00278	0.00176	0.63
SPL4	4.17126	4.16298	1.00	0.10366	0.03469	0.33
SPL6	21.93138	24.57927	1.12	0.08042	0.01139	0.14
SPL7	55.17248	111.51461	2.02	0.18246	0.13125	0.72
SPL8	10.66467	12.13463	1.14	0.00173	0.00033	0.19
SPL12	143.07002	92.20548	0.64	0.05641	0.05959	1.06
SPL13	1.59110	2.96723	1.86	0.00946	0.00263	0.28
SPL14	107.89380	219.79626	2.04	0.15572	0.16646	1.07
SPL15	10.62167	4.11869	0.39	0.00437	0.00059	0.13
SPL16A	31.26297	14.88042	0.48	0.18097	0.03594	0.20
SPL16B	16.55604	13.72896	0.83	0.02281	0.00485	0.21

RPKM: Reads Per Kilobase per Million mapped reads. RE: Relative Expression.

Table S4. SBP-domain sequences and accession numbers of selected plant SPL genes for phylogenetic analysis.

Gene Name	Protein sequence of SBP domain	Accession No or Locus ID
AtSPL10	CQIDGCELDLSSSKDYHRKHRVCETHSKCPKVVVSGLERRFCQQCSRFHAVSEFDEKKRSCRKRLSHHNARRRK	AT1G27370
AtSPL11	CQIDGCELDLSSAKGYHRKHKVCEKHSKCPKVSVSGLERRFCQQCSRFHAVSEFDEKKRSCRKRLSHHNARRRK	AT1G27360
AtSPL12	CQVDNCGADLSKVKDYHRRHKVCEIHSKATTALVGGIMQRFCQQCSRFHVLEEFDEGKRSCRRRLAGHNKRRRK	AT3G60030
AtSPL13A	CLVDGCDSDFSNCREYHKRHKVCDVHSKTPVVTINGHKQRFCQQCSRFHALEEFDEGKRSCRKRLDGHNRRRRK	AT5G50570
AtSPL13B	CLVDGCDSDFSNCREYHKRHKVCDVHSKTPVVTINGHKQRFCQQCSRFHALEEFDEGKRSCRKRLDGHNRRRRK	AT5G50670
AtSPL14	CQVDNCTEDLSHAKDYHRRHKVCEVHSKATKALVGKQMQRFCQQCSRFHLLSEFDEGKRSCRRRLAGHNRRRRK	AT1G20980
AtSPL15	CQVEGCRMDLSNVKAYYSRHKVCCIHSKSSKVIVSGLHQRFCQQCSRFHQLSEFDLEKRSCRRRLACHNERRRK	AT3G57920
AtSPL1	CQVENCEADLSKVKDYHRRHKVCEMHSKATSATVGGILQRFCQQCSRFHLLQEFDEGKRSCRRRLAGHNKRRRK	AT2G47070
AtSPL2	CQVEGCNLDLSSAKDYHRKHRICENHSKFPKVVVSGVERRFCQQCSRFHCLSEFDEKKRSCRRRLSDHNARRRK	AT5G43270
AtSPL3	CQVESCTADMSKAKQYHKRHKVCQFHAKAPHVRISGLHQRFCQQCSRFHALSEFDEAKRSCRRRLAGHNERRRK	AT2G33810
AtSPL4	CQVDRCTADMKEAKLYHRRHKVCEVHAKASSVFLSGLNQRFCQQCSRFHDLQEFDEAKRSCRRRLAGHNERRRK	AT1G53160
AtSPL5	CQVDRCTVNLTEAKQYYRRHRVCEVHAKASAATVAGVRQRFCQQCSRFHELPEFDEAKRSCRRRLAGHNERRRK	AT3G15270
AtSPL6	CQVYGCSKDLSSSKDYHKRHRVCEAHSKTSVVIVNGLEQRFCQQCSRFHFLSEFDDGKRSCRRRLAGHNERRRK	AT5G18830
AtSPL7	CQVPDCEADISELKGYHKRHRVCLRCATASFVVLDGENKRYCQQCGKFHLLPDFDEGKRSCRRKLERHNNRRKR	AT5G18830
AtSPL8	CQAEGCNADLSHAKHYHRRHKVCEFHSKASTVVAAGLSQRFCQQCSRFHLLSEFDNGKRSCRKRLADHNRRRRK	AT1G02065
AtSPL9	CQVEGCGMDLTNAKGYYSRHRVCGVHSKTPKVTVAGIEQRFCQQCSRFHQLPEFDLEKRSCRRRLAGHNERRRK	AT2G42200
OsSPL10	CQAEGCKADLSGAKHYHRRHKVCEYHAKASVVAASGKQQRFCQQCSRFHVLTEFDEAKRSCRKRLAEHNRRRRK	LOC_Os06g44860
OsSPL11	CQVEGCGLELGGYKEYYRKHRVCEPHTKCLRVVVAGQDRRFCQQCSRFHAPSEFDQEKRSCRRRLSDHNARRRK	LOC_Os06g45310
OsSPL12	CQVEGCKVDLSSAREYHRKHKVCEAHSKAPKVIVSGLERRFCQQCSRFHGLAEFDQKKKSCRRRLSDHNARRRK	LOC_Os06g49010
OsSPL13	CQVERCGVDLSEAGRYNRRHKVCQTHSKEPVVLVAGLRQRFCQQCSRFHELTEFDDAKRSCRRRLAGHNERRRK	LOC_Os07g32170
OsSPL14	CQVEGCGADLSGIKNYYCRHKVCFMHSKAPRVVVAGLEQRFCQQCSRFHLLPEFDQGKRSCRRRLAGHNERRRR	LOC_Os08g39890
OsSPL15	CQVDDCRADLTNAKDYHRRHKVCEIHGKTTKALVGNQMQRFCQQCSRFHPLSEFDEGKRSCRRRLAGHNRRRRK	LOC_Os08g40260
OsSPL16	CAVDGCKEDLSKCRDYHRRHKVCEAHSKTPLVVVSGREMRFCQQCSRFHLLQEFDEAKRSCRKRLDGHNRRRRK	LOC_Os08g41940
OsSPL17	GGSGGGGGGGGGDDVHGRHKVCYMHAKEPIVVVAGLEQRFCQQCSRFHQLPEFDQEKKSCRRRLAGHNERRRK	

OsSPL18	CAVDGCKADLSKHRDYHRRHKVCEPHSKTPVVVVSGREMRFCQQCSRFHLLGEFDEAKRSCRKRLDGHNRRRRK	
OsSPL1	CQVDGCTVNLSSARDYNKRHKVCEVHTKSGVVRIKNVEHRFCQQCSRFHFLQEFDEGKKSCRSRLAQHNRRRRK	LOC_Os01g18850
OsSPL2	CSVEGCAADLSKCVRDYHRRHKVCEAHSKTAVVTVAGQQQRFCQQCSRFHLLGEFDEEKRSCRKRLDGHNKRRRK	LOC_Os01g69830
OsSPL3	CQVEGCNVDLSSAKPYHRKHRVCEPHSKTLKVIVAGLERRFCQQCSRFHGLAEFDQKKRSCRRRLHDHNARRRK	LOC_Os02g04680
OsSPL4	CQVEGCGVELVGVKDYHRKHRVCEAHSKFPRVVVAGQERRFCQQCSRFHALSEFDQKKRSCRRRLYDHNARRRK	LOC_Os02g07780
OsSPL5	CQAEGCKADLSAAKHYHRRHKVCDFHAKAAAVLAAGKQQRFCQQCSRFHVLAEFDEAKRSCRKRLTEHNRRRRK	LOC_Os02g08070
OsSPL6	CQVEGCTADLTGVRDYHRRHKVCEMHAKATTAVVGNTVQRFCQQCSRFHPLQEFDEGKRSCRRRLAGHNRRRRK	LOC_Os03g61760
OsSPL7	CQVEGCDITLQGVKEYHRRHKVCEVHAKAPRVVVHGTEQRFCQQCSRFHVLAEFDDAKKSCRRRLAGHNERRRR	LOC_Os04g46580
OsSPL8	CQAEGCKADLSSAKRYHRRHKVCEHHSKAPVVVTAGGLHQRFCQQCSRFHLLDEFDDAKKSCRKRLADHNRRRRK	LOC_Os04g56170
OsSPL9	CQVPGCEADIRELKGYHRRHRVCLRCAHAAAVMLDGVQKRYCQQCGKFHILLDFDEDKRSCRRKLERHNKRRRR	LOC_Os05g33810
MnSPL1	CQVEDCGADLSSAKDYHRRHKVCEMHSKACKALVGNVLQRFCQQCSRFHVLQEFDEGKRSCRRRLAGHNKRRRKT	Morus013868
MnSPL2	CQVEGCNLDLSSAKDYHRKHRICESHSKSPRVIVGGVERRFCQQCSRFHGLSEFDEKKRSCRRRLSDHNARRRKP	Morus015493
MnSPL3	CQAGRGGADPSDAKRSHRRHKVCEVHSKAPVVLVAGLRQRFCQQCSRFHEVSEFDEAKRSCRRRLAGHNERRRKS	Morus009607
MnSPL4	CQADNCSVDLTDSKQYHRRHKICEFHAKATVVLVNGLQQRFCQQCSRFHGLAEFDDTKRSCRRRLAGHNERRRKS	Morus014488
MnSPL5	CQAEKCAADLSDAKQYHRRHKVCEHHAKAQVVLVAGVRQRFCQQCSRFHELSEFDETRRSCRRRLAGHNERRRKS	Morus010322
MnSPL6	CQVHGCNMDLTSSKDYHKKHRVCDVHSKTAKVIVNGIEQRFCQQCSRFHMLAEFDDGKRSCRRRLAGHNERRRKP	Morus026457
MnSPL7	CQVPTCGADIRELKGYHRRHRVCLRCANAGTVVIEGVNKRYCQQCGKFHVSSDFDEGKRSCRRKLERHNNRRRRK	Morus011281
MnSPL8	CQAEGCNADLSHAKHYHRRHKVCEFHSKASTVVAAGLTQRFCQQCSRFHLLSEFDNGKRSCRKRLADHNRRRRKT	Morus021788
MnSPL10	CQVEGCHVALVNAKDYHRRHKVCAMHSKAPKVVVLGLEQRFCQQCSRFHVLSEFEESKRSCKRRLAGHNERRRKS	Morus021787
MnSPL12	CQVEDCRADLTNAKDYHRRHKVCDMHSKASKALVGSIMQRFCQQCSRFHVLQEFDEGKRSCRRRLAGHNRRRRKT	Morus015493
MnSPL13	CLVDGCNADLSNCRDYHRRHKVCELHSKTPQVTIGGHKQRFCQQCSRFHSLEEFDEGKRSCRKRLDGHNRRRRKP	Morus010123
MnSPL14	CQVDNCKEDLSNAKDYHRRHKVCELHSKSTKALVAQQMQRFCQQCSRFHPLSEFDEGKRSCRRRLAGHNRRRRKT	Morus024784
MnSPL15	CQVEGCKVDLSDAKAYYSRHKVCGMHSKSPKVIVAGLEQRFCQQCSRFHQLPEFDQGKRSCRRRLAGHNERRRKP	Morus018032
MnSPL16A	CLVDGCKADLSSCRDYHRRHRVCEKHSKTPIVTVKGEEKRFCQQCSRFHSLGEFDEVKRSCRKRLDGHNRRRRKS	Morus010792
MnSPL16B	CLVDGCKSDLSKCRDYHRRHKVCELHSKTPRVTIGGQELRFCQQCSRFHSLVEFDEEKRSCRKRLDGHNRRRRKP	Morus017456
MdSBP1	CQVERCGADLVDAKRYYRRHKVCEFHSKAAVVIVSGIQQRFCQQCSRFHELIEFDEAKRSCRRRLAGHNERRRKSS	MDP0000861601

MdSBP2	CQVDNCKEDLSNAKDYHRRHKVCEVHSKSTRALVAKQMQRFCQQCSRFHPLSEFDEGKRSCRRRLAGHNRRRRKTQ	MDP0000149339
MdSBP3	CLVDGCNSDLSTCRDYHRRHKVCELHSKTPQVTINGNKQRFCQQCSRFHAPEEFDEGKRSCRKRLDGHNRRRRKPQ	MDP0000263766
MdSBP4	CQVYGCNKDLSSCKDYHKRHKVCEVHSKTAKVIINGIEQRFCQQCSRFHLLGEFDDGKRSCRKRLAGHNERRRKPQ	MDP0000146640
MdSBP5	CQAEGCAADLSHSKHYHRRHKVCEFHSKASTVIANGLTQRFCQQCSRFHLLSEFDNGKRSCRKRLADHNRRRRKTQ	MDP0000803116
MdSBP6	CQVEGCHVALLNAKEYHRRHKVCAMHSKASRVTVLGQDQRFCQQCSRFHVVSEFDESKRSCRRRLAGHNERRRKSS	MDP0000262141
MdSBP7	CLVDGCRADLSRCREYHRRHRVCELHSKAPVVVVRGEQKRFCQQCSRFHSLVDFDKVKRSCRKRLNGHNQRRRKPK	MDP0000170630
MdSBP8	CQADRCTADLSEEKQYHRKHKVCDLHSKSQVVLVSGLHQRFCQQCSRFHQLPEFDDTKRSCRRRLAGHNERRRKNP	MDP0000865739
MdSBP9	CQAERCGADLVDAKRYHRRHKVCEFHSKAAVVIVSGTRQRFCQQCSRFHELIEFDEAKRSCRRRLAGHNERRRKSS	MDP0000158607
MdSBP10	CQVHGCNMDLTFSKDYHKRHRVCDAHSKTAVVIVNGIKQRFCQQCSRFHLLAEFDDVKRSCRRRLAGHNLRRRKPQ	MDP0000171877
MdSBP11	CQVHGCNMDLTFSKDYHKRHRVCDAHSKTAVVIVNGIKQRFCQQCSRFHLLAEFDDVKRSCRRRLAGHNLRRRKPQ	MDP0000246046
MdSBP12	CQVEGCNLDLSSVKDYHRKHRICANHSKSPKVVVDGVERRFCQQCSRFHGLSEFDENKRSCRRRLSDHNARRRKPQ	MDP0000155354
MdSBP13	CQVEDCKADLSNAKDYHRRHKVCDMHSKATKAVVGNVLQRFCQQCSRFHGLQEFDEGKRSCRRRLAGHNRRRRKTH	MDP0000919693
MdSBP14	CQVEDCKADLSNAKDYHRRHKVCAMHSKATKALVGSVMQRFCQQCSRFHALQEFDEGKRSCRRRLAGHNRRRRKTH	MDP0000919694
MdSBP15	CQVEDCKADLSNAKDYHRRHKVCDMHSKATKALVGNVMQRFCQQCSRFHALQEFDEGKRSCRRRLAGHNRRRRKTN	MDP0000180408
MdSBP16	CQVEDCKADLSNAKDYHRRHKVCDMHSKATKAPVGNVLQRFCQQCSRFHVLQEFDEGRRSCRRRLAGHNRRRRKTH	MDP0000180409
MdSBP17	CQVPSCGIDIKELKGYHRRHRVCLACANAITVVIDGETKRYCQQCGKFHVLPDFDEGKRSCRRKLERHNNRRRRKP	MDP0000271587
MdSBP18	CQVEGCQVDLSDAKAYYSRHKVCGLHSKTPTVIVAGLEQRFCQQCSRFHLLPEFDQGKRSCRRRLAGHNERRRKPQ	MDP0000297978
MdSBP19	CLVDGCNCDLSTCRDYHRRHKVCELHSKTPQVTINGNKQRFCQQCSRFHALEEFDQGKRSCRKRLDGHNRRRRKPQ	MDP0000210138
MdSBP20	CQVYGCNKDLSSYKDYHKRHKVCEVHSKTAKVIVNGIEQRFCQQCSRFHLLGEFDDGKRSCRRRLAGHNERRRKPQ	MDP0000589558
MdSBP21	CQVHGCNMDLTFSKDYHKRHRVCDAHSKTAVVIVNGIEQRFCQQCSRFHLLAEFDDVKRSCRKRLAGHNLRRRKPL	MDP0000193702
MdSBP22	CQVHGCNMDLTFSKDYHKRHRVCDAHSKTAVVIVNGIEQRFCQQCSRFHLLAEFDDVKRSCRKRLAGHNLRRRKPL	MDP0000119458
MdSBP23	CQVEGCNLDLSSAKDYHRKHRICSNHSKSPKVVVDGVELRFCQQCSRFHGLSDFDENKRSCRKRLSDHNARRRKPQ	MDP0000249364
MdSBP24	CLVDGCNSDLSKCRDYHRRHKVCELHSKTSKVTIKGQERRFCQQCSRFHSLVEFDEGKRSCRRRLDGHNKRRRKPQ	MDP0000176265
MdSBP25	CQVPSCGVDIKELKGYHRRHRVCLACANASTVDIDGESKRYCQQCGKFHVLSDFDEGKRSCRRKLERHNNRRRRKP	MDP0000195461
MdSBP26	CLVDDCRADLSRCREYHRRHRVCELHSKTPVVVVKGEQKRFCQQCSRVHSLVEFDDGKKSCRKRLNGHNQRRRKPK	MDP0000142582
MdSBP27	CQADRCTADLSDEKKYHRKHKVCDLHSKSQVVLVSGLHQRFCQQCSRFHEXSEFDDTKRSCRSRLSGHNERRRKNP	MDP0000255162

PtSPL1	CQVEDCRADLSNAKDYHRRHKVCDVHSKASMALVGNVMQRFCQQCSRFHVLQEFDEGKRSCRRRLAGHNKRRRK	Potri.010G154000
PtSPL2	CQVDNCKEDLSKAKDYHRRHKVCQVHSKATKALVGKQMQRFCQQCSRFHPLTEFDEGKRSCRRRLAGHNRRRRK	Potri.002G002400
PtSPL3	CQVPSCEADISELKGYHRRHRVCLGCANATAVVLDGETKRYCQQCGKFHVLSDFDEGKRSCRRKLERHNNRRRRKP	Potri.010G026200
PtSPL4	CQVPGCETDISELKGYHRRHKVCLRCATATAVVLDEQTKRYCQQCGKFHVLSDFDEGKRSCRRKLERHNNRRRRKP	Potri.008G197000
PtSPL5	CQVEDCRADLSNAKDYHRRHKVCNAHSKASKALVGNVMQRFCQQCSRFHVLQEFDEGKRSCRRRLAGHNKRRRK	Potri.008G098600
PtSPL6	CQVEDCGVDLSNAKDYHRRHKVCEMHSKASKALVGNAMQRFCQQCSRFHVLQEFDEGKRSCRRRLAGHNKRRRK	Potri.014G114300
PtSPL7	CQVEDCGVDLSNAKDYHRRHKVCEMHSKASKALVGNVMQRFCQQCSRFHVLQEFDEGKRSCRRRLAGHNKRRRK	Potri.002G188700
PtSPL8	CQVEGCHVALVNAKGYHRRHKVCEMHSKAAKVIVLGLEQRFCQQCSRFHVVSEFDDAKRSCRRRLAGHNERRRK	Potri.002G142400
PtSPL9	CQVDNCKENLTTAKDYHRRHKVCEVHSKATKALVGKQMQRFCQQCSRFHPLTEFDEGKRSCRRRLAGHNRRRRK	Potri.005G258700
PtSPL11	CQVEGCNLDLSSAKDYHRKHRVCESHSKCPKVIVAGLERRFCQQCSRFHGLSEFDEKKKSCRRRLSDHNARRRK	Potri.003G172600
PtSPL12	CQVYGCNKDLSSSKDYHKRHKVCEVHSKTPQVIVDGNEQRFCQQCSRFHLLVDFDDGKRSCRKRLAGHNERRRKP	Potri.008G097900
PtSPL13	CQVYDCNKDLSSSKDYHKRHKVCEVHTKTPQVIVNGNEQRFCQQCSRFHLLVEFDDGKRSCRKRLAGHNERRRKP	Potri.010G154300
PtSPL14	CLVDGCNSDLSACRDYHRRHKVCELHSKTPQVTVGGQKQRFCQQCSRFHSLEEFDEGKRSCRKRLDGHNRRRRK	Potri.015G098900
PtSPL15	CLVDGCNSDLSTCRDYHRRHKVCELHSKTPQVTIGGQKQRFCQQCSRFHSLEEFDEGKRSCRKRLDGHNRRRRK	Potri.012G100700
PtSPL16	CQADNCTSDLADAKRYHRRHKVCEFHAKAPFAPVNGLQQRFCQQCSRFHDLSEFDDSKRSCRRRLAGHNERRRK	Potri.011G055900
PtSPL17	CQVEGCKVDLSDAKTYYSRHKVCSMHSKSPRVIVAGLVQRFCQQCSRFHLLPEFDQGKRSCRRRLAGHNERRRKP	Potri.016G048500
PtSPL18	CLVDGCTSDLSKCRDYHRRHKVCEFHSKSSQVFIKGQEQRFCQQCSRFHSLGEFDEGKRSCRKRLDGHNRRRRKP	Potri.001G058600
PtSPL19	CQVEGCNLDLSSAKDYHRKHRVCESHSKCQKVIVAGLERRFCQQCSRFHGLSEFDEKKKSCRRRLSDHNARRRK	Potri.001G055900
PtSPL20	CQVEKCGANLTDAKRYHRRHKVCEVHAKSPAVVVAGLRQRFCQQCSRFHELVEFDETKRSCRRRLAGHNERRRK	Potri.001G398200
PtSPL21	CQAEGCNADLTHAKHYHRRHKVCEFHSKASTVIAAGLTQRFCQQCSRFHILSEFDNGKRSCRKRLADHNRRRRK	Potri.002G142200
PtSPL22	CLVDGCTSDLTKCRDYHRRHKVCELHSKSRQVFIKGQEQRFCQQCSRFHSLGEFDEGKRSCRKRLDGHNRRRRK	Potri.003G169400
PtSPL23	CQVKNCTTDMTDAKRYHKRHKVCEFHAKASSVLVNGVEQRFCQQCSRFHDLSEFDDSKRSCRRRLAGHNERRRK	Potri.004G046700
PtSPL24	CQVEKCTANLTDAKQYHRRHKVCGHHAKAQVVLVAGIRQRFCQQCSRFHELSEFDETKRSCRRRLAGHNERRRK	Potri.007G138800
PtSPL25	CQVEKCGANLTDAKRYHRRHKVCEVHAKSPAVVVAGLRQRFCQQCSRFHELLEFDETKRSCRRRLAGHNERRRK	Potri.011G116800
PtSPL26	CQAEGCNADLTHAKHYHRRHKVCEFHSKASTVIAAGLTQRFCQQCSRFHLLSEFDNGKRSCRKRLADHNRRRRK	Potri.014G057700
PtSPL27	CQVEGCHVALLNAKDYHRRHKVCEMHSKAPKVIVLGLEQRFCQQCSRFHVVSEFDDAKRSCRRRLAGHNERRRK	Potri.014G057800

PtSPL28	CQVYDCNKDLSSSKEYHKRHKVCEVHSRTAKVIVNGIEQRFCQQCSRFHLLAEFDDGKRSCRKRLAGHNERRRKP	Potri.015G060400
PtSPL29	CQVEGCNLDLKSAKDYHRRHRICEKHSKSPKVIVAGMERRFCQQCSRFHELSEFDDKKRSCRRRLSDHNARRRRP	Potri.018G149900
